# Overlap in the diagnostic criteria of frontotemporal dementia syndromes with parkinsonism

**DOI:** 10.1177/13872877251316804

**Published:** 2025-02-16

**Authors:** Sami Heikkinen, Kasper Katisko, Annakaisa Haapasalo, Anne Portaankorva, Päivi Hartikainen, Eino Solje

**Affiliations:** 1 Institute of Clinical Medicine – Neurology, University of Eastern Finland, Kuopio, Finland; 2 A.I. Virtanen Institute for Molecular Sciences, University of Eastern Finland, Kuopio, Finland; 3 Research Unit of Clinical Neuroscience, Neurology, University of Oulu, Oulu, Finland; 4 Clinical Neurosciences, University of Helsinki, Helsinki, Finland; 5 Neuro Center, Neurology, Kuopio University Hospital, Kuopio, Finland

**Keywords:** Alzheimer's disease, corticobasal syndrome, dementia, diagnostic criteria, frontotemporal dementia, neurodegeneration, parkinsonism, progressive supranuclear palsy, tauopathy

## Abstract

**Background:**

Differentiating neurodegenerative diseases can be difficult in the clinical setting. This study examines the overlap of diagnostic criteria between frontotemporal dementia (FTD) syndromes with parkinsonism [e.g., corticobasal syndrome (CBS), progressive supranuclear palsy (PSP), behavioral variant frontotemporal dementia (bvFTD)] and Parkinson's disease (PD).

**Objective:**

To explore the diagnostic overlap in patients with FTD syndromes with parkinsonism and PD.

**Methods:**

Patient records from 2751 individuals at a tertiary neurological care center were reviewed, resulting in 112 bvFTD, 38 PSP, and 15 CBS patients. Clinical features and diagnostic criteria fulfillment were assessed.

**Results:**

Significant overlap in diagnostic criteria fulfilment was found: 42 bvFTD and 22 PSP patients met possible CBS criteria, 6 bvFTD patients met possible PSP criteria, and 4 met criteria for all three conditions. Higher cerebrospinal fluid levels of phosphorylated tau and tau were observed in the bvFTD group compared to PSP (p = 0.009, p = 0.002). The Mini-Mental State Examination score also differed between bvFTD and PSP (p = 0.020), and between PSP and CBS (p = 0.047). Neuroimaging showed substantial heterogeneity.

**Conclusions:**

The study reveals significant overlap in diagnostic criteria among FTD syndromes with parkinsonism, underscoring the need for more precise diagnostic criteria. Improved biomarkers could support differential diagnosis and enhance clinical trial design. Common cerebrospinal fluid biomarkers used in Alzheimer's disease diagnostics may provide additional support in the differential diagnosis.

## Introduction

The differential diagnosis of neurodegenerative diseases can be challenging, especially with parkinsonism and frontotemporal dementia (FTD) syndromes. In clinical practice, the diagnosis is mostly based on prevailing clinical diagnostic criteria rather than specific biomarkers or definite findings. These clinical diagnostic criteria have been formed and revised by expert consensus multiple times as new information and data have emerged.

Frontotemporal lobar degeneration (FTLD) is a neuropathological term used to describe a spectrum of diseases that involve degeneration of the frontal and temporal lobes of the brain. The most common disease in this category is behavioral variant frontotemporal dementia (bvFTD), typically characterized by changes in cognition and behavior.^1^ bvFTD is the second most common degenerative cognitive disease in the working class population, after Alzheimer's disease (AD).^2^ The differential diagnosis between AD and other neurodegenerative diseases is a common problem in neurology outpatient clinic. Progressive supranuclear palsy (PSP) and corticobasal syndrome (CBS) are diseases typically presenting with parkinsonism, yet defects in cognition and behavior are also common and neuropathologically these disorders are categorized as FTLD spectrum diseases.^3^ On the other hand, up to 30% of the individuals with bvFTD present with parkinsonian symptoms.^4^ These diseases may partly share common pathological mechanisms as they are mainly classified as tauopathies or transactive response DNA binding protein 43 kDa (TDP-43) proteinopathies, which differentiates them from the most common cause of parkinsonism, idiopathic Parkinson's disease (PD), characterized by α-synucleinopathy.^5^ Another common tauopathy, AD, has partly similar pathologic characteristics. The gold standard for definite diagnosis of these diseases is neuropathological confirmation or detection of causal autosomal dominant mutations, but in clinical practice these are rarely available or feasible and the inherited forms of PSP and CBS are extremely rare.

The current clinical diagnostic criteria for bvFTD was established in 2011,^6^ for CBS in 2013,^7^ for PSP in 2017^8^ and for PD in 2015.^9^ These criteria have multiple overlapping and generally common neurological features, which could lower their specificity for accurate diagnosis of a specific disorder. In this retrospective cohort study, we evaluated the patient records of a large group of patients with relevant FTD syndrome diagnoses associated with parkinsonism to study the fulfilment of these diagnostic criteria and the potential clinical overlap between diagnoses of bvFTD, PSP, and CBS, with PD regarded as an important differential diagnosis. We also investigated whether we could retrospectively discover commonly used factors or biomarkers for better differentiation of the diseases that could further increase diagnostic specificity toward a correct diagnosis.

## Methods

**Figure 1.**
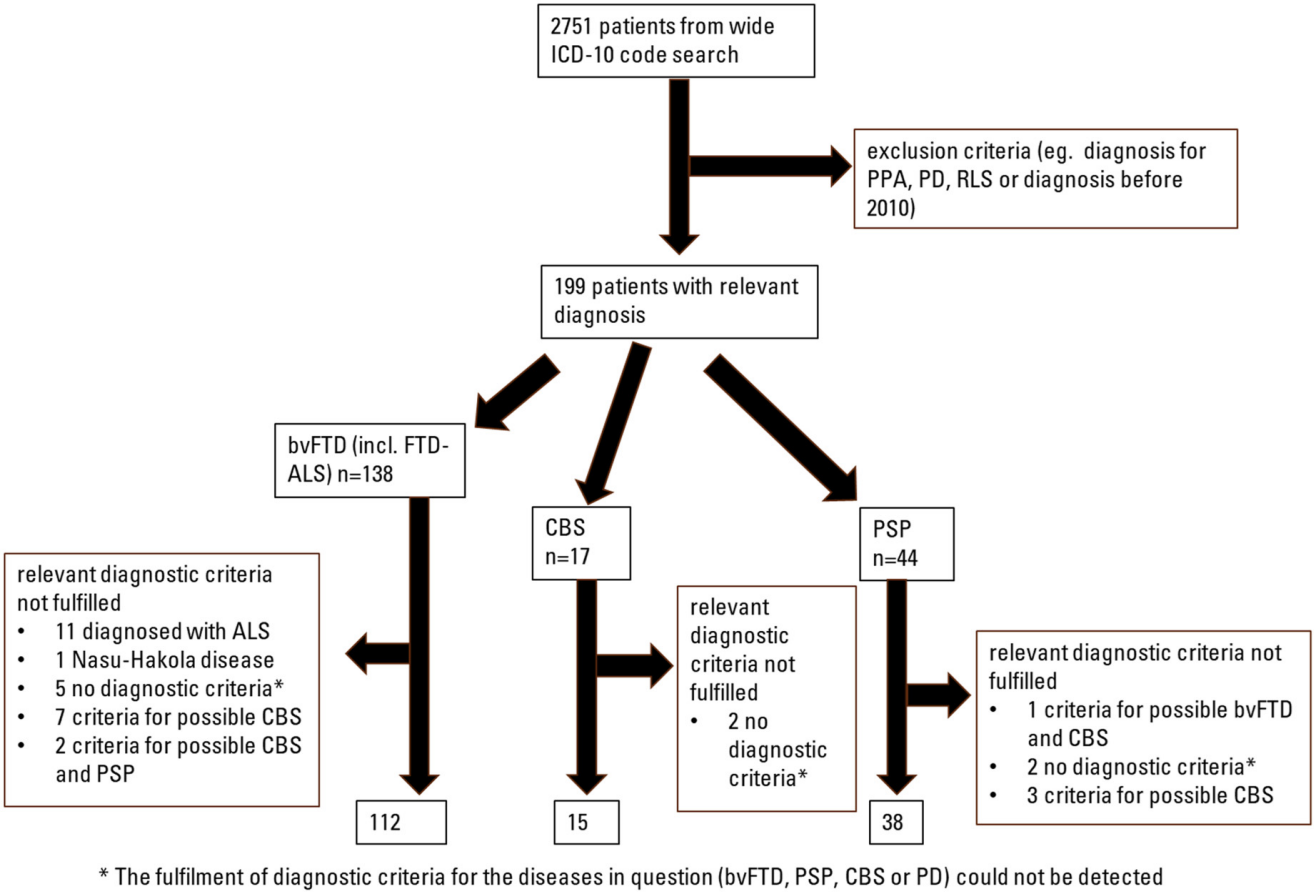
Flowchart of formation of the final cohort. ALS: amyotrophic lateral sclerosis; bvFTD: behavioral variant frontotemporal dementia; CBS: corticobasal syndrome; FTD-ALS: amyotrophic lateral sclerosis with frontotemporal dementia features; ICD-10: International Statistical Classification of Diseases and Related Health Problems; PD: Parkinson's disease; PPA: primary progressive aphasia; PSP: progressive supranuclear palsy; RLS: restless leg syndrome.

Kuopio University Hospital electronic patient register was searched with a wide range of ICD-10 -codes between January 2010 and August 2020. These codes included PD (G20) and other neurodegenerative diseases and movement disorders (G31, F02-F03, G23, G12.2, G25, G20, and F04) in order to increase the probability of acquiring all diagnoses of interest (bvFTD, PSP, CBS). A total of 2751 patients were identified using automated code search. These patient records were then individually screened by a neurologist with expertise in neurodegenerative and extrapyramidal diseases. Diagnoses not relevant for this study were excluded (such as idiopathic PD, primary progressive aphasia (PPA)-spectrum diseases, restless leg syndrome, and amyotrophic lateral sclerosis (ALS) without frontal symptoms). PPAs can have features of movement disorders present, but they exhibit never the most predominant symptom for the patient.^10^ As a result, PPA is less of a challenge in the differential diagnosis of parkinsonism and other neurodegenerative diseases, and PPA patients were thus excluded from this study. A diagnosis of ALS without frontal symptoms was determined if the patient was not reported to have cognitive or behavioral symptoms. This work resulted in the cohort of 199 patients with a relevant diagnosis (Figure 1). These patient records were then comprehensively reviewed and stored for the data of diagnostic criteria, as well as for sociodemographic factors, relevant family history of neurodegenerative diseases, and imaging data. The process of establishing the final cohort is described in Figure 1. In addition to the diagnostic criteria for bvFTD, CBS, and PSP, these individuals were assessed for fulfilment of the criteria for idiopathic PD. Demographic data as well as data of cerebrospinal fluid (CSF) biomarkers (tau, phosphorylated tau, amyloid-β (Aβ)_42_), imaging findings, and results of Mini-Mental State Examination (MMSE) were also collected and analyzed. CSF concentration of Aβ_42_ from samples taken before February 2015 were adjusted by using a constant to correct the change in the laboratory analysis procedure starting in 2015. One patient's CSF analysis was performed in 2020 using a completely different procedure compared to the other patients, resulting in its exclusion from the statistical analysis of the CSF data. The imaging findings were categorized according to the assessment of the neuroradiologist to 1) non-specific or otherwise not informative, or 2) consistent with one of the three diagnoses of interest. As this was a retrospective cohort study including only archived patient data, an ethics board approval was not acquired according with Finnish legislation.

### Statistical analyses

The data were analyzed with IBM SPSS Statistic Version 27. Demographic and clinical characteristics were summarized using descriptive statistics. Due to the non-normal distribution of the data, differences between age at diagnosis, CSF tau, phosphorylated tau, and Aβ_42_ levels, and MMSE score were compared between bvFTD, PSP and CBS groups with Mann-Whitney U test. The differences between sexes and other categorical variables were evaluated using Fisher's exact test. A *p*-value of ≤0.05 was considered as statistically significant.

## Results

Demographic information on patients is collected in Table 1.

**Table 1.** Cohort demographics and differences in the evaluated variables between the diagnostic groups.

		bvFTD (n = 112)	PSP (n = 38)	CBS (n = 15)	Difference between groups p (only p < 0.05 are displayed)
Age at diagnosis mean (SD)		67.1 (8.7)*	69.9 (7.3)	72.5 (9.3)*	*p = 0.023
Females n (%)		46 (41.1)	15 (39.5)^†^	12 (80)^†^	^†^*p *= 0.014
Patient's personal history of psychiatric illness n (%)		38 (33.9)	4 (10.5)	2 (13.3)	
Family history of extrapyramidal^‡‡^ disease n (%)		5 (4.5)	4 (10.5)	2 (13.3)	
Family history positive for cognitive disorder n (%)		49 (43.8)^‡^	4 (10.5)^‡^	3 (20)	^‡^*p *= 0.000
Family history positive for psychiatric disorder n (%)		7 (6.3)	1 (2.6)	0	
MMSE score mean (SD) at baseline assessment		22.0 (4.6)^§^	23.9 (4.6)^§,¶^	22.1 (3.2)^¶^	^§^*p *= 0.020 ^¶^*p *= 0.047
Imaging finding (MRI or PET) suggestive for the diagnosis in question		72 (64.3%)^#^	8 (21.1%)^#^	7 (46.7%)	^#^*p *= 0.000
CSF biomarker concentration mean (SD)		n = 69	n = 14	n.a. / (n = 2)	
	tau	411.5 (419.8)**	214.8 (139.7)**		**p = 0.002
	phosphorylated tau	61.2 (46.8)^††^	38.9 (18.3)^††^		^††^p = 0.009
	amyloid-β	657.3 (257.9)	664.0 (203.3)		

^‡‡^ extrapyramidal refers to motor symptoms often associated with parkinsonism, e.g., hypomimia and loss of arm swing. Each Symbol (*, †, ‡, §, ¶, #, **, ††) marks a p-value, indicating statistically significant difference between the values.

bvFTD: behavioral variant frontotemporal dementia; CBS: corticobasal syndrome; CSF: cerebrospinal fluid; MMSE: Mini-Mental State Examination; MRI: magnetic resonance imaging; PET: positron emission tomography; PSP: progressive supranuclear palsy.

Out of 138 bvFTD diagnoses (the preliminary bvFTD group), a total of 112 patients fulfilled the criteria for at least possible bvFTD in a retrospective evaluation. These 138 diagnoses included 17 patients with FTD-ALS. Of the excluded 26 patients, 11 patients were eventually diagnosed with ALS, 1 patient was diagnosed with a rare form of frontal genetic dementia (the Nasu-Hakola disease, or polycystic lipomembranous osteodysplasia with sclerosing leukoencephalopathy, OMIM 221770), 5 did not appear to fulfill the criteria of any of the diagnoses in question based on retrospective evaluation (bvFTD, PSP, CBS, or PD), 7 patients fulfilled the criteria for at least possible CBS, and 2 patients fulfilled the criteria for at least possible CBS and PSP.

In the preliminary PSP diagnosis group (n = 44), 6 patients did not fulfill the diagnostic criteria of at least possible PSP. Three patients fulfilled the criteria of at least possible CBS, 1 patient fulfilled the criteria for at least possible bvFTD and CBS, and 2 patients did not fulfill the criteria of any of the diagnoses in question.

In the preliminary CBS group (n = 17), 2 patients did not fulfill the criteria of any of the diagnoses in question.

In total, there were 4 patients who fulfilled the criteria for bvFTD, PSP, and CBS. The only case (out of the original 199 patients) fulfilling diagnostic criteria for possible PD was in the PSP group.

**Figure 2.**
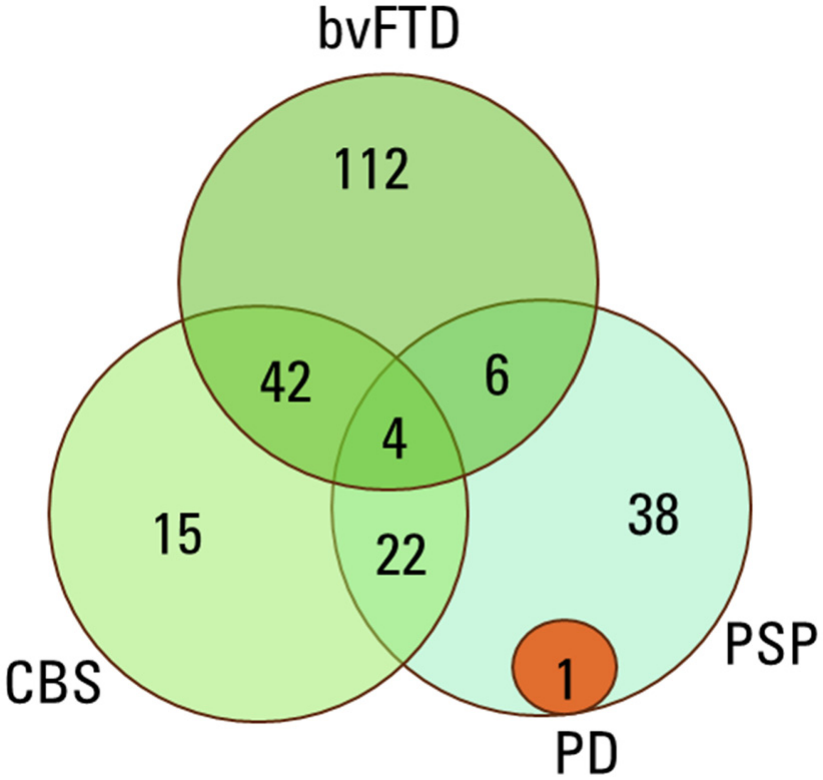
Overlap of the diagnostic criteria in the disease groups. bvFTD: behavioral variant frontotemporal dementia; CBS: corticobasal syndrome; PD: Parkinson's disease; PSP: progressive supranuclear palsy.

The final cohort used for further analyses included 165 patients. This cohort included patients with a diagnosis of interest as well as documentation for the fulfilment of the diagnostic criteria for the disease in question. The overlap of the diagnostic criteria between the diagnostic groups is visualized in Figure 2.

CSF biomarker data were available for 71 bvFTD patients, 14 PSP patients, and 2 CBS patients. The available biomarkers were the levels of tau, phosphorylated tau, and Aβ_42_ since these biomarkers are routinely used in the tertiary center in the diagnostic process of cognitive diseases. CSF was taken before the diagnosis was set. The levels of phosphorylated tau (*p *= 0.009, U = 273.5) and tau (*p *= 0.002, U = 235.5) were significantly higher in the bvFTD group compared to PSP group. The levels of Aβ_42_ did not show significant differences between the bvFTD and PSP groups (*p *= 0.657, U = 534.5). The data from CBS group were omitted due to small group size.

The imaging reports from all patients were reviewed. In total, 161 patients were scanned before the diagnosis was set. Most of the structural imaging was done with magnetic resonance imaging (MRI, 148 patients, i.e., 89.7% of the cohort). Only 13 patients were imaged with computed tomography (CT). Two patients did not have information regarding structural imaging or imaging was not available. Functional imaging (positron emission tomography, PET, or single photon emission tomography, SPECT) was performed on 77 patients before diagnosis, one patient did not have information regarding functional imaging. In the bvFTD group, the imaging finding was consistent with bvFTD in 58 patients (51.8%) and non-specific in 51 patients (45.5%). The imaging report of 1 bvFTD patient was suggestive of PSP. According to PET/SPECT in the bvFTD group, the report was consistent with bvFTD in 36 patients (32.1%) and non-specific in 29 patients. The functional imaging finding was suggestive of CBS in 3 bvFTD patients. PET/SPECT report was not available for 44 patients in the bvFTD group. In the PSP diagnosis group, MRI report was consistent with PSP in 8 patients (21.1%), non-specific in 27 patients (71.1%), and indicative of bvFTD in 1 patient. Functional imaging data were unspecific for 11 PSP diagnoses and unavailable for 27 patients. In the CBS diagnosis group, structural imaging indicated CBS in 5 patients (33.3%), was non-specific for 9 patients (60.0%), and suggestive of PSP in 1 patient. Functional imaging report was available for 7 CBS patients. The report was consistent with CBS in 5 patients (33.3% of the final CBS group) and non-specific in 2 patients (13.3% of the final CBS group).

The baseline MMSE score at the time of diagnosis was available for all 112 bvFTD patients, 32 PSP patients, and 14 CBS patients. The mean MMSE score was 22.03 (standard deviation, SD 4.565) in bvFTD group, 23.91 (SD 4.582) in the PSP group, and 22.07 (SD 3.174) in the CBS group. There was a difference in the mean MMSE score between the bvFTD and PSP groups (*p *= 0.020, U = 2215.0), as well as between PSP and CBS groups (*p *= 0.047, U = 141.0), where the PSP group scored significantly higher than both bvFTD and CBS groups.

Neuropathological information was available for 8 cases. 1 patient had PSP type pathological finding (FTLD-tau) with widespread tufted astrocytes, and 3 patients had TDP-43 predominant proteinopathy consistent with FTLD-TDP. 1 patient was suspected of having a rare form of FTLD (fused in sarcoma [FUS] or ubiquitin-proteasome system [UPS]) though no additional staining was performed. One patient had low grade amyloidosis with no aggregation, and vascular ischemic findings. Two patients had inconclusive findings and additional staining was to be performed but ultimately no pathological diagnosis was available to be found.

## Discussion

In this retrospective cohort study, we evaluated the diagnostic criteria of FTD syndromes associated with parkinsonism and compared them to diagnostic criteria of PD to assess their clinical overlap in a real-life patient population. As PSP and CBS are increasingly being recognized as FTLD-associated syndromes, it is important to report new data regarding the challenges of differential diagnosis. According to a large multinational study published in 2023, the incidence of FTLD-associated syndromes seems to be higher than previously thought.^11^

For the diagnoses of interest, the specificity of the current diagnostic criteria appears to be suboptimal. This is acknowledged in the diagnostic criteria of PSP and CBS, as they both include phenotypes of the other diseases (e.g., there are criteria for a possible PSP-CBS syndrome in both the PSP and also CBS criteria). The overlapping diagnostic criteria and limited possibilities for differential diagnosis of these diseases are prevailing challenges in clinical neurology, and have been addressed in recent publications.^12–14^ Yet, these studies do not illuminate the overlap of diagnostic criteria fulfilment, which was the main objective of our study. The only study with criteria fulfilment overlap we were able to find produced similar results.^15^ In a multicenter study where neuropathological diagnosis was available, the specificity of the 2017 PSP diagnostic criteria was reported to be good, 86%.^16^ For bvFTD, a similar study yielded a specificity of 82% for possible bvFTD with the 2011 diagnostic criteria.^17^ We were unable to find a similarly structured study for CBS, but a recent study stated that the current criteria have a positive predictive value (PPV) of 40%.^12^ This could indicate the lowest specificity for CBS diagnostic criteria. The criteria for possible CBS appear to be the least specific, as there is more overlap with other diagnoses in our study. The weakness of current diagnostic criteria has also been noted in a recent neuropathological study,^12^ in which CBD pathology was confirmed at autopsy in only 27 out of 67 patients who met the clinical criteria for possible CBS. The neuropathology of CBS is known to be heterogeneous, as CBD pathology is found in less than half of CBS cases, and the majority of CBS cases accord with PSP or AD pathology (reviewed in Koga et al., 2022). In a Finnish neuropathological case study 10 years ago where the diagnosis was made by general neurologists, sensitivity of the clinical diagnosis for PD was 82.9%, specificity 57.8%, and for PSP 52.9% and 100%, respectively, when compared to neuropathological diagnosis.^18^ However, this study was carried out with older diagnostic criteria. The accuracy of early diagnosis in parkinsonian disorders has been suboptimal for decades even in specialist movement disorder services.^19^

Neuropathological information performed postmortem was available only for a fraction of patients (4.0% of the original 199 patients), making it unfeasible to draw strong conclusions from them. However, these results showed that even with expert assessment at the time of diagnosis, and a retrospective meticulous analysis of diagnostic criteria fulfilment, the diagnosis in vivo can be inaccurate.

According to our study, the diagnostic criteria for idiopathic PD appears to differentiate PD patients from FTD syndromes associated with parkinsonism with a minimal overlap. Idiopathic PD is the second most common neurodegenerative disease,^20^ so it is possible that the development of diagnostic criteria has benefitted from large patient populations. In a recent neuropathological case study, the current PD diagnostic criteria were also noted to be very accurate in clinical practice, with clinical diagnostic accuracy of over 90% when compared to the golden standard of neuropathological diagnosis.^21^ However, as our study did not focus on PD criteria, it is not possible to conclude that PD criteria is superior to the criteria of FTD syndromes associated with parkinsonism. PPA was not included in our analyses since it predominantly presents with language deficits, it is reasonable to claim that differential diagnosis in the case of PPA is less of a problem than in other forms of FTLD. The diagnostic criteria of ALS were not applied to the cohort either, although ALS also mainly presents with movement symptoms. ALS can be differentiated from various other neurodegenerative diseases with electroneuromyography and by observing for the presence of both upper and lower motor neuron findings.

It is worth noting that the diagnostic criteria of PSP revised in 2017 includes criteria for “suggestive of PSP”, which are used for early identification of the disease.^8^ These criteria were interpreted as insufficient, as their fulfilment was high among all patients (80/165, 48.5%) in our final cohort.

An interesting detail in our findings was the predominance of female patients in CBS group compared to PSP group (80% versus 39.5%). This is in contrast to a Swiss epidemiological study, which reported that the gender difference was close to 50/50 in females and males and that there was only a slight dominance towards females in CBS,^22^ though this was not reported to be statistically significant. The small number of CBS patients in our cohort limits the value of this finding.

The challenge with the current criteria is that they are composed of features common to multiple conditions. For example, frontal or executive cognitive symptoms are a part bvFTD, PSP and CBS criteria. Also, a minor motor finding may not be caused by a neurodegenerative condition at all, as posttraumatic, musculoskeletal and rheumatic conditions can impede movement or make the classification of a symptom ambiguous. There are features in the criteria which provide great specificity toward a specific diagnosis (e.g., vertical eye movement abnormalities for PSP, alien hand syndrome for CBS), but reduce the sensitivity of criteria as these clinical details are rarely the only finding. The criteria could be improved, if new biomarker (e.g., genetic, imaging or fluid) data was included in the criteria. This requires novel research and a lengthy validation.

There was a significant difference between the levels of CSF tau and phosphorylated tau between PSP and bvFTD patients, as bvFTD patients had higher levels in both. No difference could be found regarding the Aβ_42_ levels. This is an interesting finding, as according to a review article from 2014 there were no significant differences in these biomarkers between different parkinsonian syndromes.^23^ However, assessment of CSF markers could differentiate various parkinsonian syndromes when multiple biomarkers were used as panel.^24^ The reason for the difference in these protein levels is unknown, but it is noteworthy that the proteinopathy in PSP and CBS have a different molecular classification compared to bvFTD.^25^ As these biomarkers are commonly used in the differential diagnosis of AD, they can be readily utilized to aid in the differential diagnosis of FTD spectrum disorders and parkinsonism. It is possible that with further studies and validation these AD biomarkers can also be included in the diagnostic criteria of other neurodegenerative diseases.

At the moment, there are no biomarkers for distinguishing bvFTD, PSP and CBS in routine clinical practice. However, the field is advancing rapidly, and blood-based biomarkers would provide an easily accessible way to increase diagnostic accuracy. Recently, a study utilizing the analysis of plasma extracellular vesicle tau isoforms reported good performance for distinguishing PSP from bvFTD, as well as other FTLD syndromes and healthy controls.^26^

There was also a significant difference between the baseline MMSE score between the groups, as PSP group had higher points compared to bvFTD and CBS groups. The analysis method used did not allow for inclusion of covariates, such as age or sex, but these parameters did not show significant differences between the two groups. The reason for this difference is not known. The result should be interpreted carefully, as MMSE is known to be insensitive to the cognitive changes of PSP.^3^ However, it can be seen as a signal that common cognitive screening tools developed for AD can possibly have utility in the differential diagnosis of other diseases as well.

Neuroimaging reports were found to be extremely heterogeneous, and the imaging findings were often non-specific in relation to the eventual diagnosis. This highlights the limitations in both structural and functional neuroimaging tools when considering the differential diagnostics of these parkinsonism related disorders, warranting more studies aiming to increase the diagnostic specificity. There is evidence that volumetric image measurements and analysis can provide additional confidence in the differential diagnosis of these disorders.^27^

The strengths of this study are the comprehensively and systematically acquired and assessed clinical data in a highly specialized center performed by a single physician to reduce investigator-related variability. In the Finnish healthcare records, all the further clinical data from primary, secondary, and tertiary health care can be obtained from a single source, enabling the comprehensive follow-up data analyses. The main weaknesses of this study are the retrospective nature of the data and scarcity of neuropathological confirmation of various diagnoses. Furthermore, the sizes of separate disease subgroups were relatively small, limiting the statistical comparisons.

In conclusion, a significant overlap of diagnostic criteria fulfilment was observed in the diagnostic criteria of different syndromes with parkinsonism in our cohort. This could be interpreted to be limiting the specificity of clinical diagnosis in current clinical practice. The diagnostic criteria for PD clearly differentiates patients from other parkinsonisms, whereas the diagnostic criteria for possible CBS are the least specific. This is a result of shared diagnostic criteria items, and it could be improved upon if other data was included in the criteria (e.g., biomarker data). The development of disease-specific therapies may only begin with the possibility to utilize a well-defined patient group with specific accurate diagnoses. This highlights the urgent need for better patient stratification and diagnostic tools.
